# Duct Metamaterial Muffler with Composite Acoustic Porous Media: Acoustic Optimization via Periodic Arrangement, Particle Swarm Optimization and Experimental Validation

**DOI:** 10.3390/ma18214873

**Published:** 2025-10-24

**Authors:** Ziyi Liu, An Wang, Chi Cai, Xiao Wang, Qiyuan Fan, Bin Huang, Chengwen Liu, Yizhe Huang

**Affiliations:** 1 Hubei Key Laboratory of Modern Manufacturing Quality Engineering, School of Mechanical Engineering, Hubei University of Technology, Wuhan 430068, China; 102410144@hbut.edu.cn (Z.L.); 102500010@hbut.edu.cn (A.W.); 102500042@hbut.edu.cn (C.C.); 102310162@hbut.edu.cn (X.W.); 102310147@hbut.edu.cn (Q.F.); 102110149@hbut.edu.cn (B.H.); 2 State Key Laboratory of Digital Manufacturing Equipment and Technology, Huazhong University of Science and Technology, Wuhan 430074, China; 3 Dongfeng Liuzhou Motor Co., Ltd., Liuzhou 545005, China

**Keywords:** acoustic metamaterial, porous material, air duct muffler, layout design, PSO algorithm

## Abstract

This study proposes a composite acoustic porous duct metamaterial muffler composed of a perforated tortuous channel and an externally wrapped porous layer, integrating both structural resonance and material damping effects. Theoretical models for the perforated plate, tortuous channel, and porous material were established, and analytical formulas for the total acoustic impedance and transmission loss of the composite structure were derived. Finite element simulations verified the accuracy of the models. A systematic parametric study was then performed on the effects of porous material type, thickness, and width on acoustic performance, showing that polyester fiber achieves the best results at a thickness of 30 mm and a width of 5 mm. Further analysis of periodic distribution modes revealed that axial periodic arrangement significantly enhances the peak noise attenuation, radial periodic arrangement broadens the effective bandwidth, and multi-frequency parallel configurations further expand the operating range. Considering practical duct conditions, a single-layer multi-cell array was constructed, and its modal excitation mechanism was clarified. By employing the Particle Swarm Optimization (PSO) algorithm for multi-parameter optimization, the average transmission loss was improved from 26.493 dB to 29.686 dB, corresponding to an increase of approximately 12.05%. Finally, physical samples were fabricated via 3D printing, and four-sensor impedance tube experiments confirmed good agreement among theoretical, numerical, and experimental results. The composite structure exhibited an average experimental transmission loss of 24.599 dB, outperforming the configuration without porous material. Overall, this work highlights substantial scientific and practical advances in sound energy dissipation mechanisms, structural optimization design, and engineering applicability, providing an effective approach for broadband and high-efficiency duct noise reduction.

## 1. Introduction

Noise pollution has become one of the major environmental problems in modern society. In ventilation and air-conditioning systems, industrial pipelines, and transportation ducts, aerodynamic noise threatens environmental comfort and equipment stability. Traditional mufflers, such as resistive and reactive types, show limited efficiency in the low-frequency range and suffer from large volume and complex structures. Acoustic metamaterials, with subwavelength characteristics and artificial structural design, offer a new solution for low-frequency noise control. However, single-type metamaterial structures still provide narrow bandwidth and unstable attenuation performance, restricting their engineering applicability. Therefore, coupling porous materials with metamaterial structures, by combining material damping and structural resonance, has become an effective path to improve broadband low-frequency noise reduction in practical duct systems.

Acoustic metamaterials are composite structural materials. They possess acoustic properties not found in ordinary materials, such as negative mass density, negative equivalent elastic modulus, and negative refractive index [1]. The concept of metamaterials originally came from the research field of electromagnetism. In 1968, Russian scientist Veselago [2] pointed out that, when the key physical quantities characterizing material dispersion—permeability and permittivity—are both negative, the material will exhibit negative refractive index properties. Before this theory was confirmed, the academic community only recognized positive refractive indices. After years, Smith, Pendry [3,4], and others took the lead in constructing an array structure coupled by split resonant rings and metal filaments. This structure aimed to achieve negative permeability and negative permittivity in sequence. The advent of such electromagnetic metamaterials not only broke through the limitations of traditional electromagnetic field principles but also provided theoretical support and technical paths for the formation and development of the concept of acoustic metamaterials. In the 20th century, scholars such as Liu [5,6] first introduced the new concept of acoustic metamaterials. They used small lead balls covered with silica gel as basic units. These units were inserted into epoxy resin according to the distribution pattern of square crystals, creating a locally resonant phononic crystal. This crystal could generate negative equivalent mass density and achieve the goal of manipulating large wavelengths at the microscale. Based on this, they explained the formation mechanism of elastic wave bandgaps caused by the local resonance effect. Subsequently, Fang [7] and his team designed a linear array of Helmholtz resonators, aiming to achieve negative equivalent modulus. Liang [8] and others innovatively designed the shape of resonant cavities. They constructed two-dimensional coiled acoustic metamaterials by designing the curled channels into a zigzag shape. Later, Frenzel’s [9] team innovatively proposed a 3D spiral acoustic metamaterial. On this basis, Zhang’s [10] research team further constructed a 3D-configured single-port spiral acoustic metamaterial. To explore the influence of resonator arrangement on acoustic properties, Cai [11] and others conducted acoustic studies on Helmholtz resonators with series arrays, parallel arrays, and parallel-opposed arrays. They found that different installation methods have a significant impact on acoustic transmission loss. Based on research on coiled metamaterial sound absorption structures, Huang [12] and others innovatively incorporated the design concept of internal insertion holes. This strategy provides a more feasible adjustment method for efficient sound absorption control of acoustic metamaterials in different frequency ranges.

Scholars have designed various muffler structures using optimization methods. Their goal is to achieve the best noise reduction performance of metamaterial pipelines. To explore the noise reduction performance of membrane-type metamaterial coupled pipeline mufflers, Wang [13] and others designed a membrane-type acoustic metamaterial muffler. It solved the problem that airflow could not flow smoothly in the channel after being covered by membranes. In 2021, Xiao [14] and others adopted a coiled scheme. They embedded four circular labyrinth resonant cavities near the airflow channel. Based on optimization via the variable cross-section method, they achieved noise reduction in the wide frequency range of 660~1200 Hz. Qi [15] and others coupled resonant cavities with spatial labyrinth microstructures. They proposed a labyrinth air duct muffler based on acoustic metamaterials. It achieved noise reduction greater than 10 dB in the wide frequency range of 230~816 Hz. Min [16] and others proposed a new type of air duct muffler. It consists of a microperforated plate with cavities of different depths arranged in parallel on its radial back. Through simulation optimization, it achieved noise reduction higher than 20 dB in the frequency range of 700~1600 Hz. Subsequently, Du [17] and others proposed a ventilation labyrinth-type acoustic metamaterial. Based on simulation optimization algorithms, its transmission loss was above 5 dB in the frequency range of 250~380 Hz, with a maximum of approximately 24 dB. Wu [18] and others designed an acoustic metamaterial for pipeline fan noise. It uses a series coiled structure to achieve ultra-thinness, controlling the first four orders of tonal noise. Through theory, experiments, and CFD/FEM simulations, it was confirmed that the noise reduction ranged from 3.03 to 11.64 dB, with an impact on ventilation efficiency of approximately 2.6%. This provides a compact noise reduction solution.

Porous materials have poor low-frequency sound absorption performance. However, composite structures can improve this. To explore the composite acoustic effect of resonant cavities and porous materials, Li [19] and others combined perforated plate resonators with porous materials. This resulted in a metamaterial with composite acoustic impedance, improving sound absorption performance in the mid/low-frequency range of 200~1600 Hz. Within a limited 80 mm space, the average sound absorption coefficient predicted by the acousto-electrical analogy analysis model was greater than 0.89. Liu [20] and others used a Helmholtz resonator as the backing cavity. They connected it to a porous material with progressively increasing perforation sizes. The total thickness of the structure was 120 mm, aiming to achieve broadband sound absorption at mid/low frequencies. They verified this by theoretically applying a surface correction model, realizing the surface transfer matrix method. Li [21] and others used multi-cavity coupled resonators combined with a melamine foam porous material, forming an acoustic structure with a thickness of 70 mm. They optimized the structure using the G-A method, achieving good sound absorption in the frequency range of 255~1600 Hz. Li [22] and others focused on the composite of melamine sponge and scatterer structures. They adopted topology optimization combined with genetic algorithm (TOGA) to optimize the distribution of internal scatterers in a 5 cm thick melamine sponge. The goal was to increase the average sound absorption coefficient in the 200~1600 Hz range to 0.8. They also studied the influence of multiple factors and verified its effectiveness. By using a compact structure composed of coiled channel resonance with mesopores and multi-scale gradient porous materials, Li [23] and others analyzed its acoustic performance based on the transfer matrix method and finite element simulation. Due to the mesopore resonance of the coiled channels, the coupling effect between multi-scale porous materials and coiled channels, and energy trapping between mesopores and the porous material matrix, the broadband absorption of porous materials can be shifted to lower frequencies through coiled channels and enhanced. A composite structure with a width of 70 mm and a thickness of 100 mm was designed through parallel coupling, achieving efficient sound absorption in the 240~3000 Hz frequency band. Zhang [24] and others constructed an acoustic metamaterial with a porous lining by adding a porous material lining to the coiled air channel, enhancing the dissipation of sound energy. Based on the theoretical model of dual porosity and impedance transfer method, efficient sound absorption was achieved in the frequency range of 405~879 Hz. By using periodic acoustic metamaterial resonators embedded in a porous layer, Zhu [25] and others realized broadband absorption of low-frequency sound waves. A sound absorption coefficient greater than 0.8 was obtained in the 180~550 Hz range, which can be controlled by adjusting the inner radius of the structure. Guo [26] and others proposed a layered porous acoustic metamaterial, combining resonant cavities with porous materials. Broadband sound absorption was achieved through synergistic energy dissipation and impedance matching. Experiments showed an average sound absorption coefficient of 0.77, with 73% of α points exceeding 0.8. Ultra-broadband characteristics can be achieved after optimization. In addition, to address the challenge of low-frequency broadband sound absorption, Liu [27] and others proposed a honeycomb-type gradient perforated porous acoustic metamaterial. Combined with embedded necks, they modeled it using dual porosity theory and the transfer matrix method. It achieved a sound absorption bandwidth of 58.5% at 265 Hz with a thickness of 52 mm. After paralleling seven units, the sound absorption coefficient exceeded 0.9, providing a new solution for low-frequency noise reduction.

Current research shows that acoustic metamaterials and porous materials exhibit good synergistic effects in low-frequency broadband noise control. They have significant application potential, especially in achieving broadband noise reduction in the low-frequency range. However, the coupling mechanism in composite structures remains unclear, and the influence of structural and material parameters on overall acoustic performance still lacks systematic clarification. Moreover, most reported studies focus primarily on simulation or simplified theoretical models, while the correlation between experimentally measurable parameters (such as material porosity, flow resistivity, and cavity geometry) and the predicted noise reduction performance is still insufficiently explored. In addition, efficient multi-parameter collaborative optimization strategies integrating both experimental validation and numerical modeling are still rare.

Therefore, based on the design of a metamaterial muffler with perforated and tortuous characteristics, this paper introduces externally wrapped acoustic porous materials to construct composite noise reduction units. Focusing on broadband noise attenuation, the study conducts structural modeling, parameter analysis, optimization, and experimental verification. Furthermore, optimization algorithms are incorporated to improve performance through arrangement-mode and structural-parameter optimization. By establishing a closed-loop framework of theoretical modeling–simulation analysis–experimental validation, this work bridges the gap between experimental parameter understanding and literature models, providing a new optimization design path for compact, efficient, and tunable low-frequency broadband mufflers.

## 2. Structural Design and Noise Reduction Characteristics of Composite Muffler

**Figure 1:**
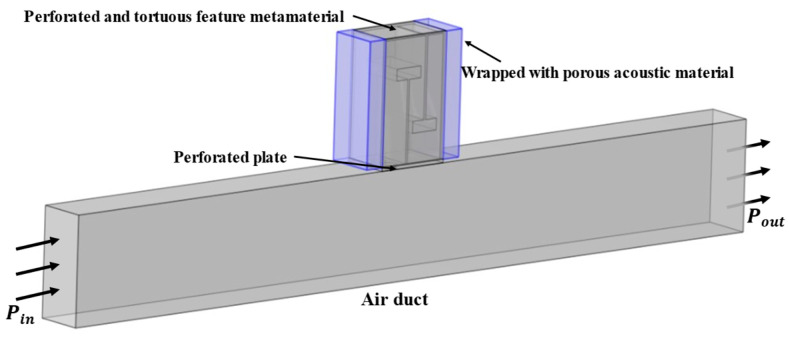
Composite acoustic metamaterial muffler for air duct.

To achieve broadband noise suppression in the mid/low-frequency range within limited space for ventilation ducts, this paper designs a new type of acoustic metamaterial structure with perforated and tortuous characteristics combined with porous materials, as shown in Figure 1. This structure is composed of a surface perforated plate and a tortuous structure [28]. Acoustic porous materials are wrapped around the front and rear of this structure to form a composite acoustic metamaterial muffler, which is installed on the noise reduction duct.

### 2.1. Theoretical Analysis of Perforated and Tortuous Metamaterial

The acoustic metamaterial structure with perforated and tortuous characteristics is mainly composed of a perforated panel and multi-stage tortuous channels. When sound waves propagate through it, multiple reflections and thermoviscous losses occur, thus effectively attenuating mid/low-frequency sound energy.

A perforated plate contains multiple tiny holes, which can be approximately equivalent to a parallel structure of multiple short tubes [29,30]. According to the principle of acoustoelectric analogy [31], the characteristic impedance of the perforated plate can be expressed as:

(1) $$Z_{p}=R_{p}+jX_{P}$$

In the equation, *Rₚ* is the acoustic resistance, determined by viscous friction; *Xₚ* is the acoustic reactance, generated by the mass effect inside the holes; their expressions are, respectively, as follows:

(2) $$R_{p}=\frac{32\eta h}{d^{2}\sigma }$$

(3) $$X_{P}=\frac{\omega \rho_{0}\left(h+2\delta \right)}{\sigma }$$

Among them, *h* is the thickness of the perforated plate, *d* is the hole diameter, *σ* is the perforation rate, *η* is the aerodynamic viscosity of air, *δ* is the end correction length (usually 0.85d), and *ω* is the angular frequency.

After elaborating on the acoustic characteristic impedance *Zₚ* of the perforated plate structure, we further analyze the acoustic properties of the air medium in the tortuous channels. To reveal the energy loss mechanism of sound wave propagation in microscale channels, an equivalent complex medium model is introduced to correct the viscous and thermal dissipation of air. The effective complex density *ρₑ_q_* and complex bulk modulus *Kₑ_q_* of the air medium are, respectively, expressed as [32]:

(4) $$\rho_{eq}=\rho_{0}\left[1+\frac{F\left(\omega \right)}{j\omega }\right]$$

(5) $$K_{eq}=\gamma P_{0}{\left[1+\frac{G\left(\omega \right)}{j\omega }\right]}^{-1}$$

In the equation, *ρ*_0_ is the density of air under standard conditions, with a value of 1.22 kg/m^3^; *γ* is the specific heat ratio, with a value of 1.4; *P*_0_ is the standard atmospheric pressure. *F*(*ω*) and *G*(*ω*) are the viscous and thermal dissipation correction functions, respectively, which are specifically expressed as follows:

(6) $$F\left(\omega \right)=\frac{2}{r_{eq}}\sqrt{\frac{\eta }{\rho_{0}\omega }}$$

(7) $$G\left(\omega \right)=\frac{2}{r_{eq}}\sqrt{\frac{\kappa }{\rho_{0}c_{p}\omega }}$$

In the equation, *κ* is the thermal conductivity of air, with a value of 0.0262 W/(m·K); *cₚ* is the specific heat capacity at constant pressure, with a value of 1005 J/(kg·K); *r_eq_* is the equivalent radius of the channel (usually taken as half of the channel height).

Using the above complex medium parameters, the complex propagation constant *k* and characteristic acoustic impedance *Z_c_* of sound waves in the channel can be further obtained:

(8) $$k=\omega \sqrt{\frac{\rho_{eq}}{K_{eq}}}$$

(9) $$Z_{c}=\sqrt{\rho_{eq}\cdot K_{eq}}$$

Among them, the complex propagation constant k characterizes the attenuation and phase delay of sound waves, while the characteristic acoustic impedance *Z_c_* reflects the response characteristics of the local channel to sound waves.

For the tortuous channel structure, it can be equivalently divided into multiple short straight channel segments, and the transfer matrix method is used for acoustic impedance recursion. Each segment has a length of *l_i_*, and its transfer matrix can be expressed as:

(10) $$T_{i}=\left[\begin{array}{cc} \cos(kl_{i}) & jZ_{c}\sin(kl_{i})\\ j\sin(kl_{i})/Z_{c} & \cos(kl_{i}) \end{array}\right]$$

Considering that the tortuous channel can be decomposed into several short straight channel segments, the transfer matrix method can be introduced to describe the relationship between sound pressure and volume flow velocity at both ends of each channel segment. Its expression is as follows:

(11) $$\left[\begin{array}{c} p_{in}\\ u_{in} \end{array}\right]=T_{i}\left[\begin{array}{c} p_{out}\\ u_{out} \end{array}\right]$$

Since the tortuous channel is composed of multiple folded segments, each segment can be regarded as a series unit, and the overall modeling adopts a segment-by-segment recursive approach to solve the input impedance. Assuming the end is rigidly closed, the impedance of the last segment is infinite. According to the acoustic transmission theory, the expression for the input impedance of any *i*-th segment can be obtained by recursing from the rear to the front as follows:

(12) $$Z_{i}^{in}=Z_{c}\cdot \frac{Z_{i+1}^{in}+jZ_{c}\tan(kl_{i})}{Z_{c}+jZ_{i+1}^{in}\tan(kl_{i})}$$

By recursing segment by segment from the end segment to the first segment, the equivalent input impedance of the entire tortuous cavity channel can finally be obtained:

(13) $$Z_{c}^{in}=Z_{1}^{in}$$

Since the sound wave propagation path passes through the perforated plate and enters the tortuous cavity in sequence, the two form a series connection in the acoustic model. Its total input impedance *Z_total_* can be expressed as:

(14) $$Z_{total}=Z_{p}+Z_{c}^{in}$$

The characteristic acoustic impedance of air, *Z*_0_, is:

(15) $$Z_{0}=\rho_{0}c_{0}$$

Sound waves incident on the structure from the free field are partially absorbed through impedance matching. Under the condition that both ends of the structure are in the free field, by combining Equations (14) and (15), the transmission coefficient *τ* of sound waves is as follows:

(16) τ=2Z0·Re(Ztotal)Ztotal+Z02

Finally, by substituting Equation (16) into the definition formula of acoustic transmission loss, the sound transmission loss (*STL*) of the muffler with this structure can be obtained as follows:

(17) $$STL=-10\log_{10}(\left|\tau \right|)$$

### 2.2. Theoretical Analysis of Acoustic Porous Materials

To study the propagation characteristics of sound waves in porous media, numerous scholars have constructed and proposed various macroscopic theoretical models for analysis [33]. To accurately characterize the sound absorption properties of porous materials in mid- to high-frequency acoustic behaviors, this section introduces the Johnson-Champoux-Allard (JCA) model for theoretical modeling. This model considers the viscous effects of air in the porous skeleton and heat exchange losses, and can comprehensively describe the propagation mechanism of sound waves in porous media.

In the JCA model, porous materials are equivalent to isotropic media with frequency-dependent properties. Its equivalent density *ρ*(*ω*) and equivalent bulk modulus *K*(*ω*) are, respectively, expressed as:

(18) $$\rho (\omega )=\frac{\alpha_{\infty }\rho_{0}}{\phi }\left[1+\frac{\sigma \phi }{j\omega \alpha_{\infty }\rho_{0}}(1+j\frac{4\alpha_{\infty }^{2}\rho_{0}\eta \omega }{\sigma^{2}\Lambda^{2}\phi^{2}})\right]$$

(19) $$K(\omega )=\frac{\gamma p_{0}}{\phi }{\left\{\gamma -(\gamma -1){\left[1-j\frac{8\eta }{\Lambda^{\prime 2}p_{r}\rho_{0}\omega }{\left(1+j\frac{\Lambda^{\prime 2}p_{r}p_{0}\omega }{16\eta }\right)}^{\frac{1}{2}}\right]}^{-1}\right\}}^{-1}$$

In the equation, *ω* is the angular frequency; *α*_∞_ is the tortuosity; *η* represents the viscosity coefficient; *ρ*_0_ is the air density under standard conditions; *ϕ* is the porosity; *σ* is the flow resistivity; *p*_0_ stands for the static pressure; *γ* is the specific heat ratio; *p_r_* is the Prandtl number of airs; Λ denotes the viscous characteristic length; and Λ′ represents the thermal characteristic length.

Further, the equivalent characteristic impedance *Z_dk_* and equivalent wavenumber *k_dk_* of porous materials can be solved using the above Equation (18):

(20) $$Z_{dk}=\sqrt{\rho \left(\omega \right)\cdot K\left(\omega \right)}$$

(21) $$k_{dk}=\omega \sqrt{\rho \left(\omega \right)/K\left(\omega \right)}$$

When the thickness of the porous acoustic material is *h_dk_* and its back is in close contact with an acoustic hard boundary, its surface acoustic impedance *Z_s_* can be approximately calculated as:

(22) $$Z_{s}\approx -jZ_{dk}\;\text{cot}\left(k_{dk}h_{dk}\right)$$

### 2.3. Theoretical Analysis of Acoustic Composite Mufflers

By integrating Equations (18)–(22), the surface acoustic impedance *Z_s_* of the acoustic porous material is derived. Further, based on the acoustical–electrical analogy method, the composite structure can be regarded as being composed of three parallel units. According to impedance theory, the calculation expression for the total surface acoustic impedance *Z_PZCs-all_* of this composite structure is as follows:

(23) $$Z_{\text{PZCs-all}}^{-1}=Z_{\text{s-l}}^{-1}+Z_{total}^{-1}+Z_{\text{s-r}}^{-1}$$

In the equation, *Z_s-l_* and *Z_s-r_* represent the acoustic characteristic impedances of the acoustic porous materials wrapped on the left and right sides, respectively. *Z_total_* is the result derived in Section 2.1. Finally, by modifying Equation (16) to the following Equation (24), the sound transmission loss of the composite structure muffler can be obtained.

(24) τ=2Z0·Re(ZPZCs-all)ZPZCs-all+Z02

### 2.4. Validation of Model Effectiveness

**Figure 2:**
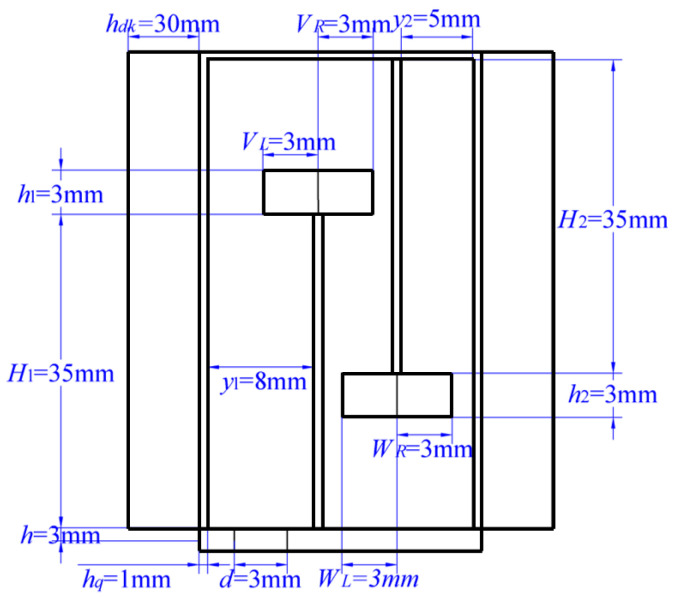
Dimensional parameters of the composite acoustic metamaterial muffler.

To verify the validity of the proposed acoustic theoretical model, a three-dimensional structural model was established and simulated using COMSOL Multiphysics 6.1. The schematic structure of the composite muffler is shown in Figure 1, and its dimensional parameters are illustrated in Figure 2. The porous layer thickness was set to *h_dk_* = 30 mm, and a polyester fiber was selected as the porous material, with parameters listed in Table 1.

**Figure 3:**
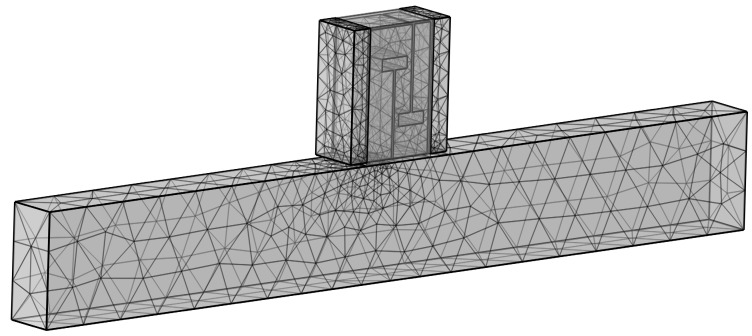
Simulation meshing diagram of the duct composite acoustic metamaterial muffler.

During the finite element simulation, the following settings for the model are required [28]:

(1) In the finite element simulation, the “acoustic–solid coupling” module and the “thermoviscous acoustics” module are used for multiphysics coupling. As sound waves enter the perforated tortuous channel from the air duct, the airflow velocity changes abruptly. This results in significant viscous and thermal losses in narrow regions. Therefore, the air in the air duct and the perforated channel is divided into the pressure acoustics domain and the thermoviscous acoustics domain, respectively. The solid structure boundaries are classified into the solid mechanics domain.
(2) The interior of the air duct and the perforated tortuous metamaterials are set as air domains. The boundaries between structures are set as hard sound field boundaries. To reduce errors caused by boundary reflection, in the pressure acoustics module, the inlet end of the air duct is set as a plane wave excitation port with a sound pressure amplitude of 1 Pa. The outlet end is set as a plane wave transmission port, as shown by *P_in_* and *P_out_* in Figure 1. Sound waves enter the composite muffler structure along the axial direction, thus achieving effective coupling.
(3) In terms of materials, the medium inside the cavity is air. Therefore, the pre-set Air material from the COMSOL material library is selected for filling. Its parameters are the default parameters of the software’s material library, and the ambient temperature is set to 20 °C.
(4) A free tetrahedral mesh is used for the air duct and muffler, and a swept mesh is applied to the acoustic porous material. During meshing, it is necessary to ensure that the maximum element size of the mesh is no more than 1/6 of the wavelength corresponding to the highest frequency. The minimum element size is no more than 1/15 of the wavelength corresponding to the lowest frequency. The approximate frequency range studied in this simulation model is 200~700 Hz [28]. The finally generated mesh is shown in Figure 3.

In COMSOL software, the sound transmission loss of the composite acoustic metamaterial muffler is as follows:

(25) $$STL=20\;\log_{10}\left(\frac{P_{in}}{P_{out}}\right)$$

In the equation, *P_in_* and *P_out_* denote the incident sound pressure and transmitted sound pressure, respectively.

**Figure 4:**
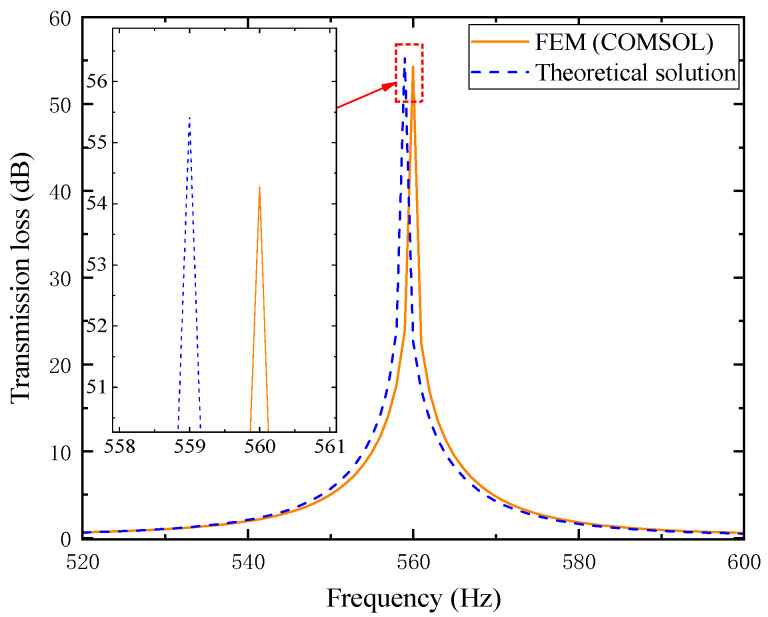
Comparison between simulation and theoretical results of the composite acoustic metamaterial muffler.

The comparison between its analytical solution and simulation results is shown in Figure 4. Wherein, the orange solid line represents the simulation values, and the blue dashed line represents the theoretical values.

In the figure, the theoretical and simulation curves agree well within the error range. The simulated resonance frequency is 560 Hz with a peak transmission loss of 54.275 dB, while the theoretical values are 559 Hz and 55.427 dB, respectively, confirming the accuracy of the analytical model.

It should be noted that the theoretical model in this study is established under several reasonable simplifications: the incident wave is assumed to be planar, the boundaries are rigid, and the propagation process is linear, without considering structural vibration, temperature non-uniformity, or boundary leakage. In addition, the perforated plate and tortuous channel are idealized as one-dimensional acoustic elements with approximate end correction and thermoviscous parameters, whereas the finite element model captures the detailed three-dimensional flow and corner effects. As a result, slight deviations may occur at high frequencies or near resonance peaks. Overall, these differences reflect limited physical approximation and geometric idealization rather than model inaccuracy. The high consistency between theoretical and FEM results confirms that the analytical model maintains excellent precision and reliability within the design frequency range.

## 3. Influence of Porous Material Parameters on Noise Reduction Performance

### 3.1. Influence of Types of Porous Materials on Noise Reduction Performance of Air Ducts

To compare the noise reduction effects of different porous materials, three common materials were selected: a polyester fiber, a rock wool, and a glass wool. The acoustic parameters of each material are shown in Table 1, Table 2, and Table 3, respectively.

**Figure 5:**
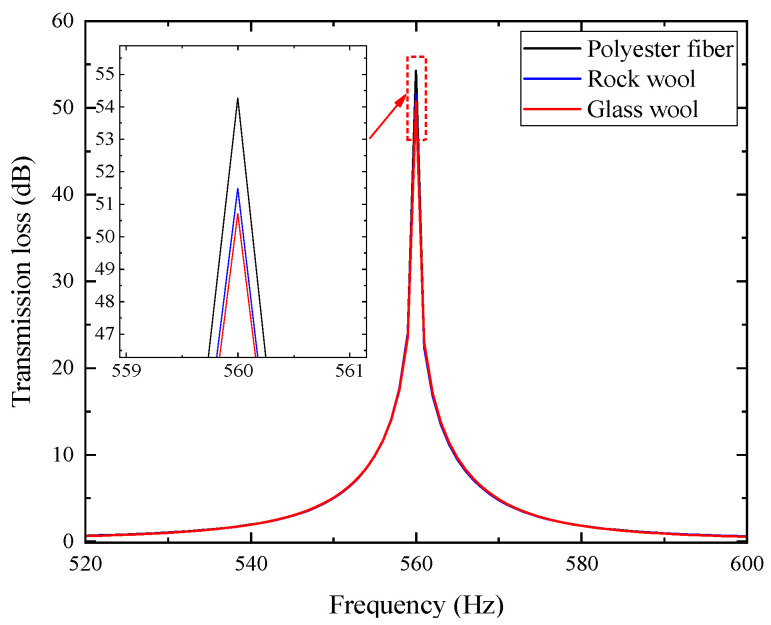
Influence of three different types of porous materials on noise reduction performance.

With the porous layer thickness unified at *h_dk_* = 30 mm and other parameters kept constant, the transmission loss curves corresponding to different porous materials are obtained, as shown in Figure 5.

In the figure, the composite mufflers using the three porous materials exhibit identical resonance frequencies, but their transmission loss peaks differ. The polyester fiber material shows the best performance with a peak value of 54.275 dB, followed by rock wool (51.486 dB) and glass wool (50.707 dB). Therefore, the polyester fiber material was adopted in subsequent studies.

### 3.2. Effect of Porous Material Thickness on Noise Reduction Performance of Air Ducts

**Figure 6:**
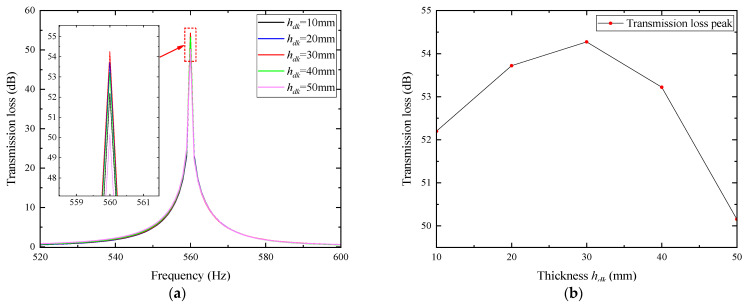
Influence of porous materials with different thicknesses on noise reduction performance: (**a**) frequency characteristics of transmission loss for porous materials with different thicknesses; (**b**) peak of transmission loss as a function of porous material thickness.

For noise reduction through coupling with acoustic porous materials, the thickness is the most direct influencing factor. Under the condition that other parameters remain constant, the thickness *h_dk_* was set to 10, 20, 30, 40, and 50 mm, and the transmission loss curves corresponding to different thicknesses were obtained, as shown in Figure 6.

In the figure, the transmission loss peak of the composite muffler increases first and then decreases with the increase in porous layer thickness, while the resonance frequency remains nearly unchanged. The maximum peak value of 54.275 dB occurs at a thickness of 30 mm.

### 3.3. Influence of Width of Porous Materials on Noise Reduction Performance of Air Ducts

**Figure 7:**
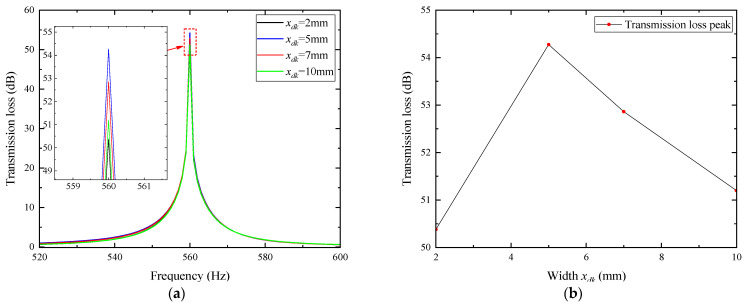
Influence of porous materials with different widths on noise reduction performance: (**a**) frequency characteristics of transmission loss for porous materials with different widths; (**b**) peak of transmission loss as a function of porous material width.

The width *x_dk_* of the porous layer, defined as its axial length along the duct, affects the overall volume and thus the acoustic performance. With other parameters kept constant, *x_dk_* was set to 2, 5, 7, and 10 mm, and the transmission loss curves at different widths were obtained, as shown in Figure 7.

In the figure, the transmission loss peak of the composite muffler increases first and then decreases with the increase in porous layer width, while the resonance frequency remains almost unchanged. The maximum peak value of 54.275 dB occurs at a width of 5 mm.

## 4. Layout Design of Perforated Tortuous Characteristic Metamaterial Mufflers

### 4.1. Influence of Two Periodic Distribution Modes on the Performance of Metamaterial Mufflers

(1) Radial periodic distribution

**Figure 8:**
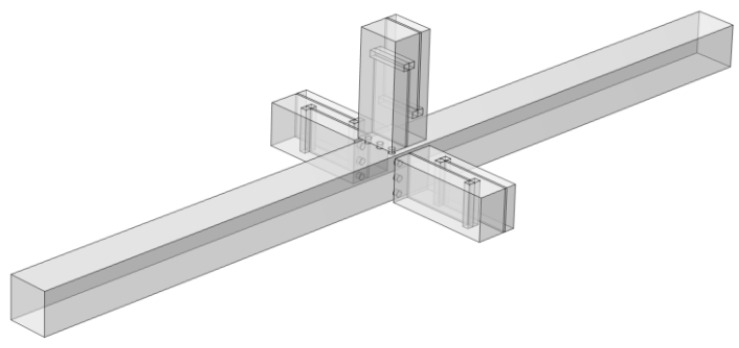
Radial periodic structure (*X* = 3).

**Figure 9:**
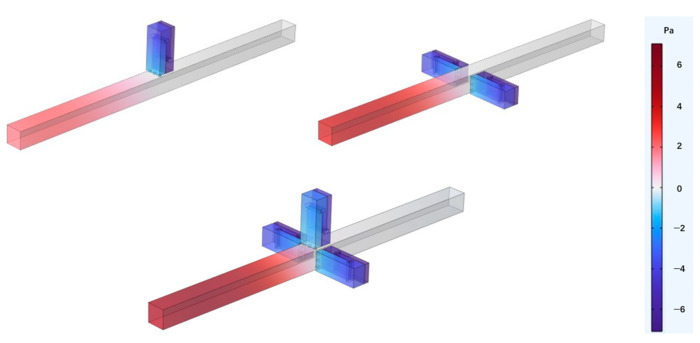
Sound pressure distributions for various radial period numbers (resonant frequency 570 Hz).

Perforated tortuous metamaterial units with identical resonance frequencies were periodically arranged along the radial direction of the duct. The number of periods *X* was set to 1, 2, and 3, with unit angles of π/2, −π/2, and 0, respectively. The corresponding sound pressure and transmission loss curves were obtained. Figure 8 shows the radial periodic arrangement of the metamaterial units, and the sound pressure distribution at 570 Hz is illustrated in Figure 9. As the sound wave enters the main duct, each cavity is successively excited. With increasing radial periodicity, the sound pressure response decreases and the attenuation bandwidth broadens, but the overall noise reduction performance does not significantly improve.

**Figure 10:**
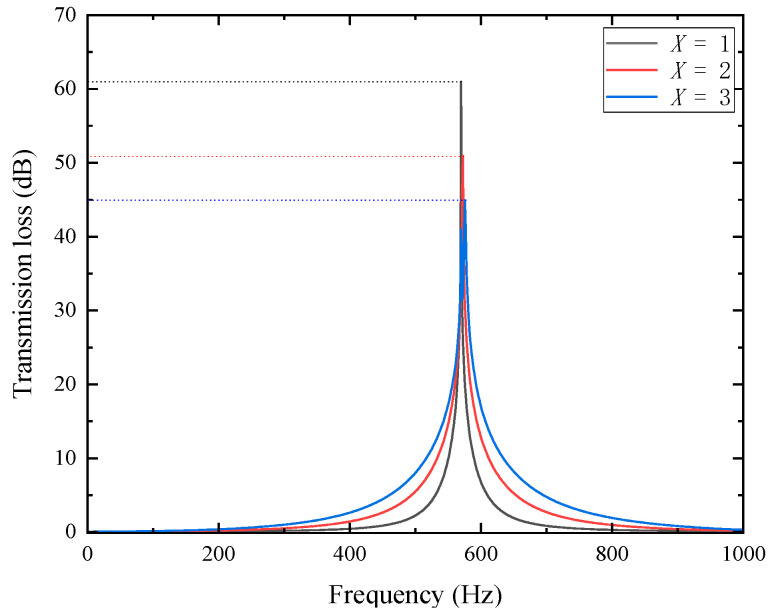
Sound transmission loss characteristics for various radial period numbers.

To verify the above inference, COMSOL simulation software was used to conduct a simulation analysis on the sound transmission loss characteristics of the metamaterials arranged periodically along the radial direction, and the results are shown in Figure 10.

Although the overall structure only exhibits one peak of sound transmission loss, compared with a single perforated tortuous metamaterial unit, the introduction of radial periodicity leads to a slight upward shift in the resonant frequency and a decrease in the peak value. The results in the figure show that when *X* = 1, the resonant frequency is 570 Hz, and the peak sound transmission loss is 60.943 dB; when *X* = 2, the resonant frequency rises to 573 Hz, and the peak sound transmission loss drops to 50.927 dB; when *X* = 3, the resonant frequency also slightly rises to 576 Hz, and the peak sound transmission loss drops to 44.907 dB. In contrast, the effective attenuation bandwidth (*TL* > 10 dB) expands markedly—from 40 Hz for *X* = 1 to 79 Hz and 118 Hz for *X* = 2 and *X* = 3. This indicates that increasing radial periodicity enhances average transmission loss and broadband attenuation, even though the resonance peak slightly weakens. The added periodic cells introduce inter-cavity coupling, which promotes distributed energy dissipation and bandwidth broadening, while minor mode splitting and phase mismatch smooth the resonance response. Hence, the structure evolves from a narrowband resonator toward a broadband absorber, representing a realistic trade-off between peak intensity and usable bandwidth.

(2) Axial periodic distribution

**Figure 11:**
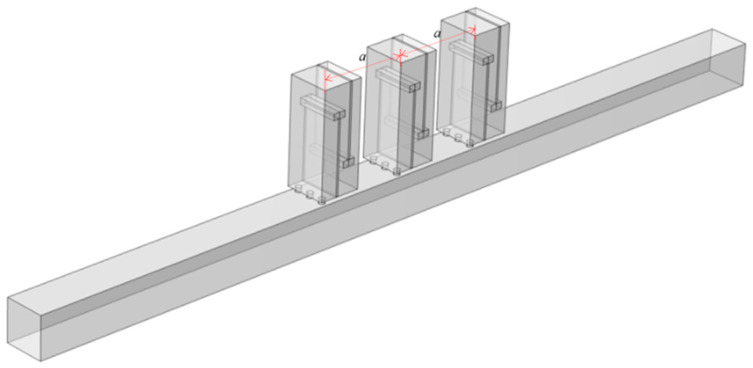
Axial periodic structure (*Y* = 3).

**Figure 12:**
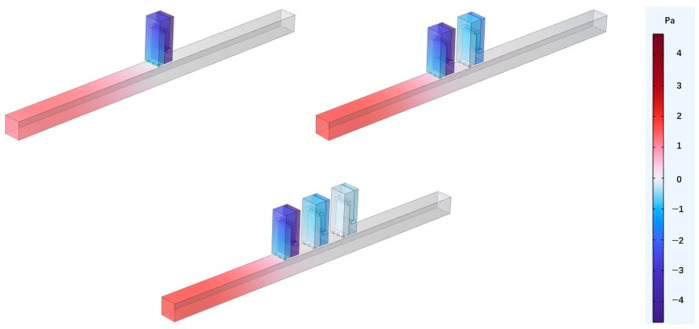
Sound pressure distributions for various axial period numbers (resonant frequency 570 Hz).

Perforated tortuous characteristic metamaterial mufflers with the same resonant frequency are periodically arranged along the axial direction of the air duct, distributed at equal intervals of *a* = 20 mm with period numbers *Y* = 1, 2, 3. The corresponding sound pressure and sound transmission loss are calculated, and the influence on the acoustic performance of the tortuous characteristic metamaterial air duct mufflers with axial periodic distribution is analyzed. Figure 11 shows the axial periodic arrangement of the units, and Figure 12 illustrates the sound pressure distribution at 570 Hz. As the sound wave propagates along the main duct, each unit is successively excited, and the acoustic energy is gradually dissipated. With an increasing number of axial periods, the sound pressure decreases and the noise reduction effect is enhanced, indicating that axial periodicity can improve transmission loss and broaden the attenuation bandwidth.

**Figure 13:**
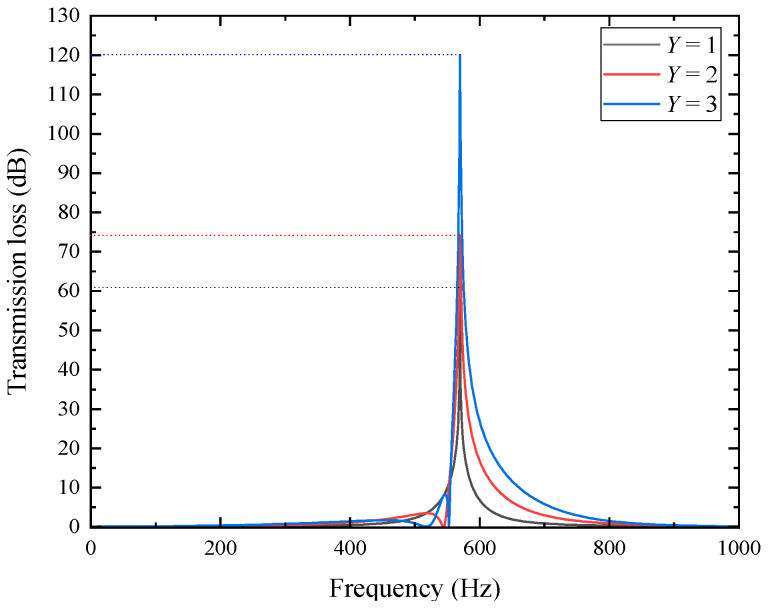
Sound transmission loss characteristics for various axial period numbers.

To verify the above inference, a simulation analysis was also conducted on the sound transmission loss characteristics of metamaterials arranged periodically along the axial direction, with the results shown in Figure 13.

In the figure, the resonance frequency remains constant at 570 Hz regardless of the axial period number, and all transmission loss curves exhibit a single-peak profile. With an increasing number of periods, the peak value rises significantly and the bandwidth expands, enhancing the overall noise reduction performance. Specifically, for *Y* = 1, 2, 3, the peak transmission losses are 60.943 dB, 74.055 dB, and 120.01 dB, and the effective bandwidths (*STL* > 10 dB) are 40 Hz, 73 Hz, and 107 Hz, respectively. These results demonstrate that increasing the axial periodicity effectively improves the overall sound attenuation and broadens the absorption bandwidth. The enhanced performance originates from the phase accumulation and constructive interference between adjacent periodic units along the axial direction, which promote multi-mode coupling and more uniform energy dissipation.

It should be noted that the transmission loss peak of approximately 120 dB shown in Figure 13 results from a narrow-band resonance under idealized conditions of plane-wave incidence and rigid-wall boundaries. In practical engineering applications, factors such as leakage, wall flexibility, and flanking transmission can significantly reduce the peak value. Therefore, this value represents only the local resonance enhancement effect in theoretical simulations, rather than the actual performance of commercial mufflers.

### 4.2. Study on Parallel Connection of Metamaterial Mufflers with Multiple Resonant Frequencies

**Figure 14:**
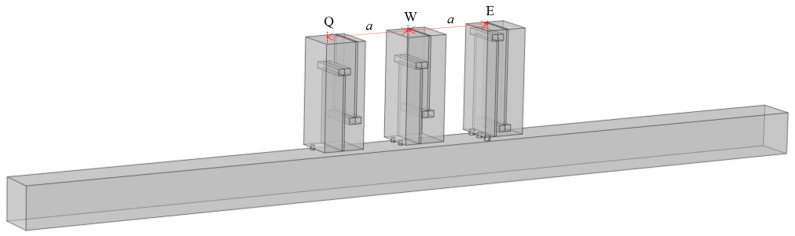
Axially parallel-connected Q, W, E metamaterial mufflers.

**Figure 15:**
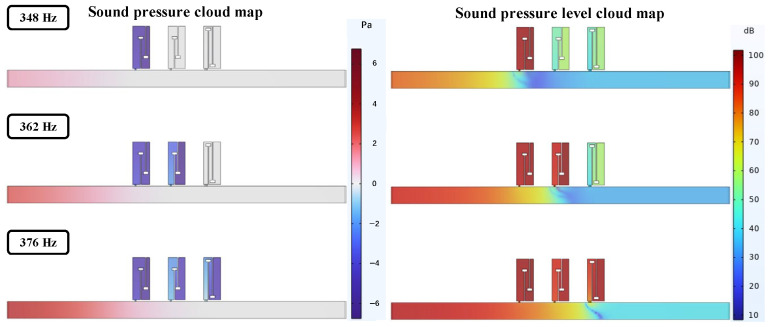
Sound pressure and sound pressure level distribution contour maps of axial three-cell metamaterial mufflers with different resonant frequencies.

Based on the previous section, axially connecting multiple perforated tortuous metamaterial mufflers with identical resonance frequencies can enhance the transmission loss peak at that frequency. On this basis, this section further investigates the effect of connecting multiple structures with different resonance frequencies on transmission loss. Through parameter optimization, three metamaterial structures with similar resonance frequencies, denoted as Q, W, and E, were designed, and their structural details and resonance frequencies are listed in Table 4. Q, W, and E are sequentially arranged in the air duct, as shown in Figure 14. Their sound pressure, sound pressure level distribution, and transmission loss curves are calculated, and the sound pressure distribution is shown in Figure 15.

**Figure 16:**
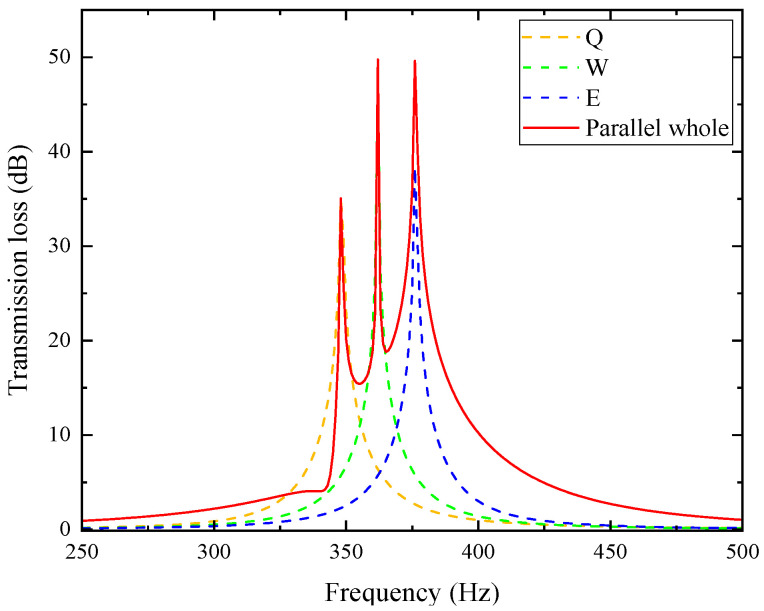
Sound transmission loss curves of three-cell axially parallel-connected mufflers.

Table 4 lists the resonant frequencies and peak sound transmission losses of single-cell metamaterials Q, W, and E. The three units were sequentially arranged along the direction of sound incidence, and their sound pressure and sound pressure level distributions are shown in the figure. The sound wave first enters unit Q, where a maximum negative pressure occurs at 348 Hz and the inlet sound pressure level drops sharply, indicating effective attenuation. Similar behavior is observed in units W and E at 362 Hz and 376 Hz, respectively. To further analyze the sound transmission loss characteristics of single cells and the combined structure, the sound transmission loss curves of the three units and the overall curve of their combination are plotted in the same figure, as shown in Figure 16. The three dashed-dot lines in the figure correspond to the respective curves of Q, W, and E, while the solid line represents the overall sound transmission loss curve of the parallel combination of the three.

In the figure, the transmission loss curve of the three parallel units exhibits three peaks at 348, 362, and 376 Hz, which are consistent with the resonance frequencies of each unit listed in Table 4, indicating a superposition effect. The corresponding peak values are 35.051 dB, 49.756 dB, and 49.622 dB, showing that the overall performance surpasses that of any single unit. Further analysis shows that the noise reduction frequency band of the parallel structure muffler with a sound transmission loss greater than 10 dB is 345~401 Hz, with a bandwidth of 56 Hz. In contrast, the noise reduction frequency bands of the single cells Q, W, and E are 340~357 Hz, 354~370 Hz, and 368~385 Hz, respectively, with bandwidths of 17 Hz, 16 Hz, and 17 Hz. After fitting the sound transmission loss curves of the three, the frequency band is 340~385 Hz with a bandwidth of 45 Hz. Thus, the parallel configuration not only enhances transmission loss but also broadens the attenuation band. Moreover, the coupling between units with different resonance frequencies alters the system’s effective mass and stiffness, causing a slight upward shift in the overall resonance frequency, like the frequency increase observed when stiffness rises in a mass–spring system.

**Figure 17:**
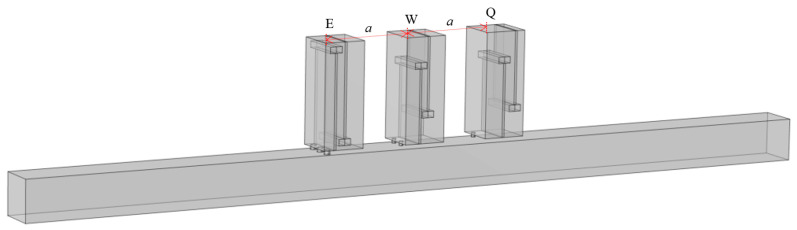
E, W, Q axially parallel-connected metamaterial mufflers.

**Figure 18:**
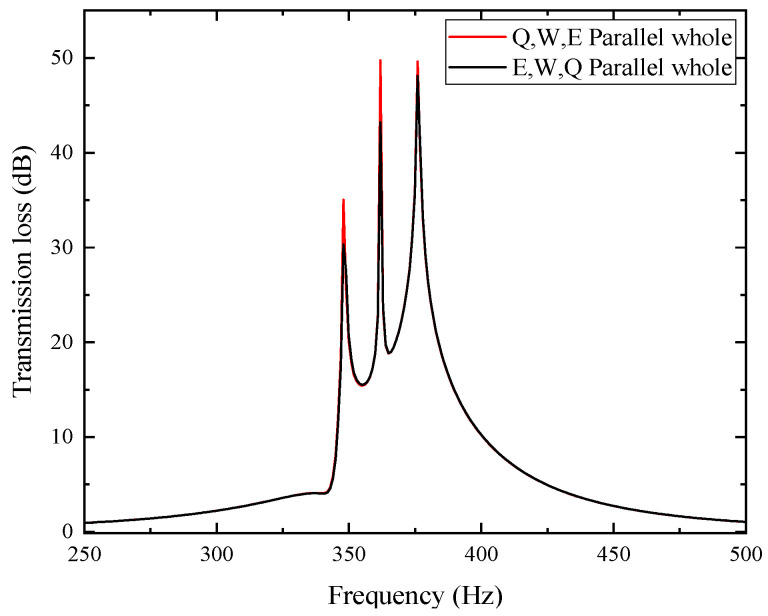
Sound transmission loss curves for two arrangement orders.

To further investigate the influence of cell arrangement order on the overall noise reduction performance, the positions of Q and E are swapped, as shown in Figure 17, so that they are no longer arranged in ascending order of resonant frequency. A comparative analysis of the sound transmission loss curves of the two arrangement modes is conducted, with the results shown in Figure 18 and the specific characteristic values listed in Table 5.

In the figure and table, the peak sound transmission loss at each resonant frequency for the Q, W, E arrangement is higher than that for the E, W, Q arrangement. Moreover, both arrangements have the same resonant frequencies, and the sound transmission loss curves of the other parts basically overlap. Therefore, the Q, W, E arrangement has better noise reduction performance. This difference primarily arises from the asymmetric coupling and directional energy dissipation among multiple resonant cells along the duct axis. When the units are arranged in ascending order of resonant frequency, the incident acoustic energy is successively dissipated through sequential excitation of each resonator, forming a cumulative energy attenuation path and a smoother impedance gradient that facilitates multi-mode coupling. In contrast, when the high-frequency unit is placed first (E, W, Q), abrupt impedance discontinuity and partial reflection occur, weakening subsequent resonance excitation and reducing overall transmission loss. It can be concluded that arranging the single cells in sequence along the axial direction of the air duct in ascending order of their resonant frequencies helps to improve the performance of the overall metamaterial muffler.

### 4.3. Analysis of Noise Reduction Characteristics of the Single-Layer Structure Model

**Figure 19:**
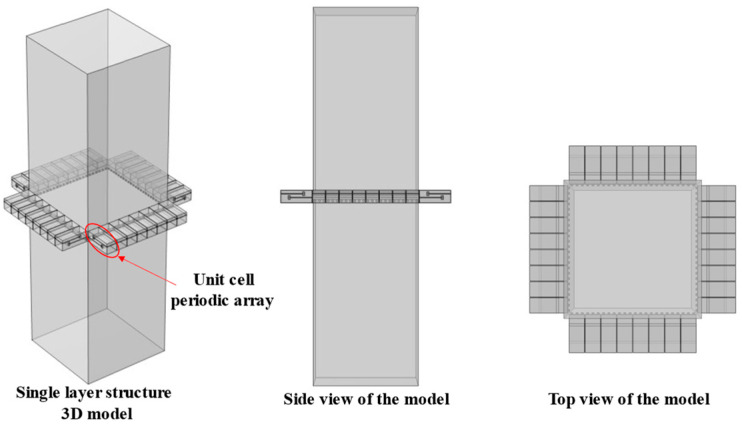
Schematic diagram of the structure of the periodic single-layer metamaterial silencer for the actual air duct.

Further considering the actual size of air conditioning ducts, to balance noise reduction performance and space utilization, single-cell structures are first arranged periodically in the radial direction, and then multi-layer periodic structures with different resonant frequencies are constructed in the axial direction to form an air duct muffler model. This section first analyzes the sound transmission loss characteristics of the single-layer metamaterial in this structure, and its 3D model is shown in Figure 19.

In the single-layer metamaterial model, 8 single-cell metamaterials are radially arrayed on each surface of the air duct, with a total of four surfaces, so each layer contains 32 single-cell metamaterials in total. Assuming there are *N* single-cell metamaterials connected in parallel (where *N* = 32 here), based on the idea of the acoustical–electrical analogy method and combined with impedance theory, the calculation formula for the total surface acoustic impedance *Z_PZC-all_* of *N* parallel-connected metamaterials is as follows:

(26) $${Z_{\text{PZC-all}}}^{-1}=\sum_{n=1}^{N}\left({Z_{\text{PZC-n}}}^{-1}\right)$$

**Figure 20:**
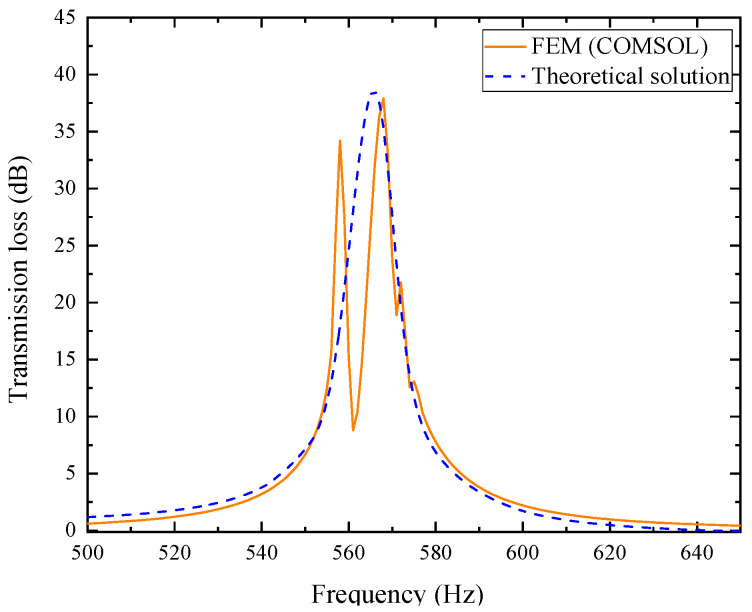
Sound transmission loss curve of the periodic single-layer metamaterial silencer for the actual air duct.

In the equation, *Z_PZC-n_* represents the acoustic characteristic impedance of each perforated tortuous characteristic acoustic metamaterial noise-reducing resonator cell. For this single-cell structure, the basic structure from Section 2.1 is still adopted. Based on the COMSOL finite element simulation method, the acoustic transmission loss of this parallel-connected noise reducer is calculated, as shown in Figure 20, where the orange-red solid line is the simulation curve and the blue dashed line is the theoretical curve.

**Figure 21:**
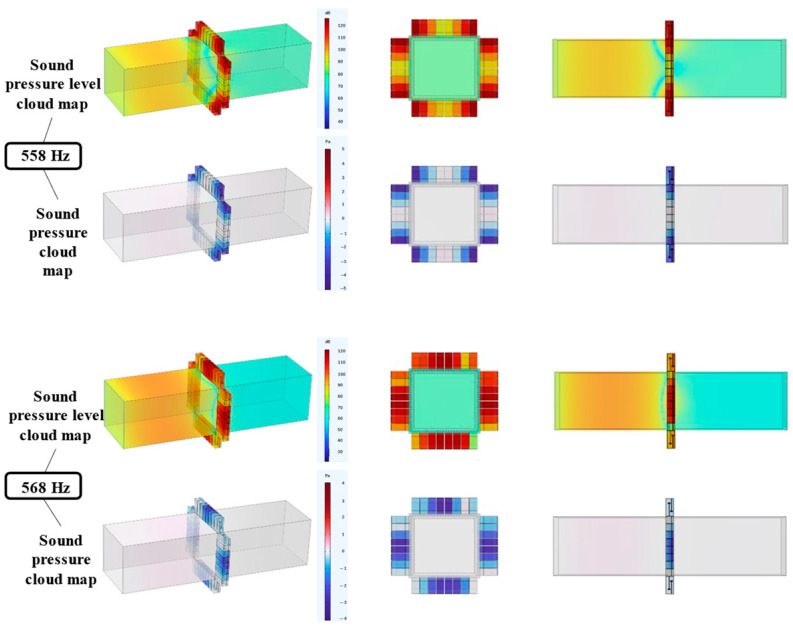
Sound pressure and sound pressure level contour maps of the periodic single-layer metamaterial muffler for the actual air duct.

In the figure, the simulation results of the multi-cell parallel single-layer duct metamaterial muffler agree well with the theoretical predictions in the 500~555 Hz and 568~650 Hz frequency ranges. However, noticeable deviations occur between 555 and 568 Hz, where the simulation curve exhibits distinct fluctuations. Two prominent transmission loss peaks appear at 558 Hz (34.206 dB) and 568 Hz (37.899 dB), with an effective attenuation bandwidth (*STL* > 10 dB) of 553~577 Hz and a width of 24 Hz, which is broader than that of the single-cell structure. The theoretical results show a peak of 38.625 dB at 566 Hz, which is close to the second resonance point in the simulation. This conforms to the law in radial periodic structures where the resonant frequency of the overall structure increases while the peak value decreases. The local deviation in this narrow frequency band is primarily associated with the multi-modal acoustic behavior induced by the rectangular cavity geometry, where local sound pressure superposition and interference lead to fluctuations in the simulation curve. Considering the sound pressure and sound pressure level amplitude contour maps of the model comprehensively, as shown in Figure 21, a more detailed analysis of the acoustic characteristics of the multi-cell parallel single-layer metamaterial muffler is conducted.

Combining Figure 20 and Figure 21, due to the rectangular shape of the air duct cavity, the propagation path of sound waves in it is more complex, and the reflection and interference effects are more obvious, resulting in the excitation of two independent resonant modes. When the sound wave frequency matches these two modes, the sound transmission loss increases significantly, forming two distinct peaks. In Figure 21, the first-order mode is excited at a frequency of 558 Hz, where the sound pressure distribution is concentrated near the four right-angle corners of the rectangular channel. The corresponding single-cell metamaterial muffler modules resonate, while the remaining areas are almost ineffective. At 568 Hz, the second-order mode is excited, with sound pressure concentrated at the center of the four surfaces; the modules in the corresponding areas resonate, while the corner areas remain inactive. Therefore, it can be concluded that the first-order mode of the metamaterial muffler is mainly excited near the rectangular corners, while the second-order mode is excited in the central regions of the four surfaces.

## 5. Optimization Design of Mufflers Based on PSO Algorithm

### 5.1. Introduction to Particle Swarm Optimization (PSO) Algorithm

Particle Swarm Optimization (PSO) is a global optimization method based on swarm intelligence, proposed by Kennedy and Eberhart in 1995 [34]. By simulating the cooperative behaviors of biological groups such as bird flocks and fish schools during foraging, this algorithm realizes global search in complex function spaces. It has advantages such as simple algorithm structure, high computational efficiency, and fast convergence rate [35].

The PSO algorithm was chosen for its favorable balance between convergence stability, accuracy, and computational efficiency when dealing with nonlinear multi-parameter acoustic optimization problems. Preliminary tests with GA and DE under identical conditions showed similar or lower convergence performance; therefore, PSO was adopted as a representative and reliable optimization framework in this study.

The PSO algorithm operates through a set of particles moving in the search space, with each particle influenced by its own historical best position *P_best_* and the historical best position of the entire swarm *G_best_* [36]. Specifically, the update formulas for the velocity *v_i_^t^* and position *x_i_^t^* of the *i*-th particle in the *t*-th generation are as follows:

(27) $$v_{i}^{t+1}=\omega v_{i}^{t}+c_{1}r_{1}(P_{best}-x_{i}^{t})+c_{2}r_{2}(G_{best}-x_{i}^{t})$$

(28) $$x_{i}^{t+1}=x_{i}^{t}+v_{i}^{t+1}$$

In the equations *ω* is the inertia weight factor; *c*_1_ and *c*_2_ are learning factors; *r*_1_ and *r*_2_ are random numbers in the interval [0, 1]; *P_best_* is the particle’s own historical best position; and *G_best_* is the historical best position of the entire swarm.

### 5.2. Structural Optimization Design of Metamaterial Mufflers

Based on the study in Section 4, a 20-layer metamaterial muffler without porous material was established for optimization. The structure was divided into four groups, each containing five layers, and optimized separately. Considering that automotive air-conditioning duct noise mainly occurs in the 300~500 Hz range—with narrower bandwidths at low frequencies and wider ones at high frequencies—the target frequency intervals for each group were set as shown in Table 6. During optimization, only individuals within the specified frequency range were retained, and the configurations with higher average transmission loss were selected. The final optimization results are summarized in Table 7.

**Figure 22:**
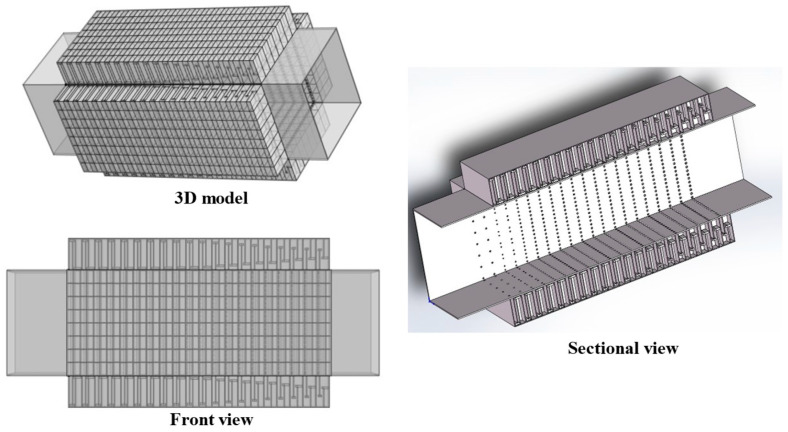
Optimized simulation model of the acoustic metamaterial muffler.

**Figure 23:**
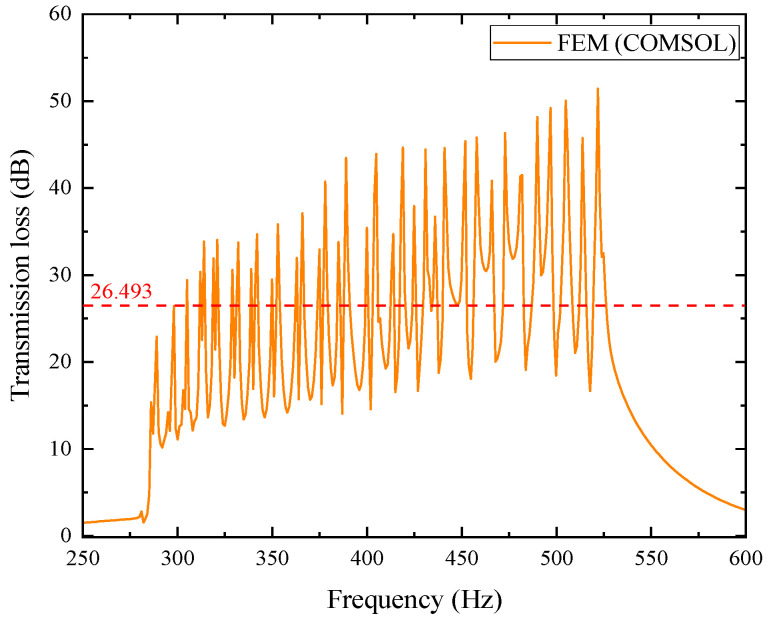
Simulation curve of sound transmission loss for the optimized acoustic metamaterial muffler.

Using the COMSOL simulation platform, a finite element model of the optimized 20-layer duct metamaterial muffler was established. The schematic diagram of this simulation model is shown in Figure 22. Its sound transmission loss was calculated, and the resulting data are presented in Figure 23.

**Figure 24:**
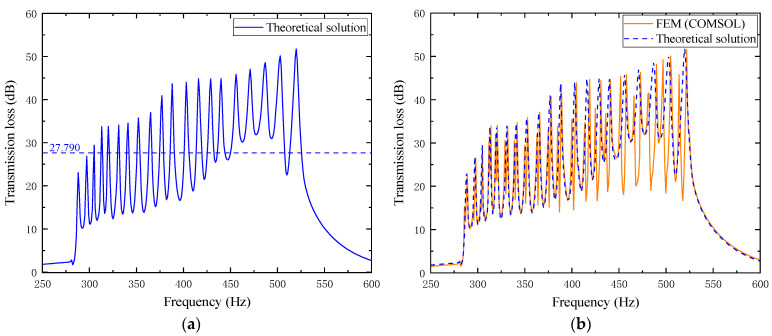
Comparison between simulation and theoretical results of the optimized acoustic metamaterial muffler: (**a**) theoretical solution; (**b**) comparison of FEM and theoretical solution.

In the figure, each layer’s transmission loss value excites second-order modes. Within the effective noise reduction frequency band of 287~550 Hz, a total of 20 pairs of transmission loss peaks with values greater than 10 dB are formed, meeting the optimization requirements. Observing the above figure, it can be found that as the frequency increases, the frequency of each pair of transmission loss peaks shows an upward trend. The average transmission loss obtained from the simulation calculation is 26.493 dB. For further investigation, the simulation results are compared with theoretical results, where the orange curve represents the simulation results and the blue curve represents the theoretical calculation results. The comparison is shown in Figure 24.

From the comparison chart of optimized simulation and theoretical results, the average transmission loss of the theoretical curve is 27.790 dB. In the mid/low-frequency range of 287~400 Hz, the curve fitting between the two is good, with an average error of 1.754% between the simulation and theory, and the second-order mode excited in each layer is not obvious. However, in the mid/high-frequency range of 400~520 Hz, the curve fitting is poor, with the average error reaching as high as 15.619%. The second-order mode excited in each layer is prominent, which has a significant impact on the average transmission loss.

### 5.3. Parameter Optimization Design of Porous Materials

Based on the previous study, a polyester fiber was selected as the porous material and applied to both sides of the metamaterial, while the optimal arrangement of the non-porous structure in the duct was determined. On this basis, the PSO algorithm was employed to optimize the thickness *h_dk_* and width *x_dk_* of the porous layer to enhance the performance of the composite metamaterial muffler. The optimization objective was the transmission loss peak *TL_max_* at the resonance frequency, with a higher value being preferable. To ensure structural rationality and manufacturability, the control ranges of the variables were defined as follows:

(29) $$s.t.\left\{\begin{array}{l} 10<h_{dk}<52\\ 2<x_{dk}<10 \end{array}\right.$$

**Figure 25:**
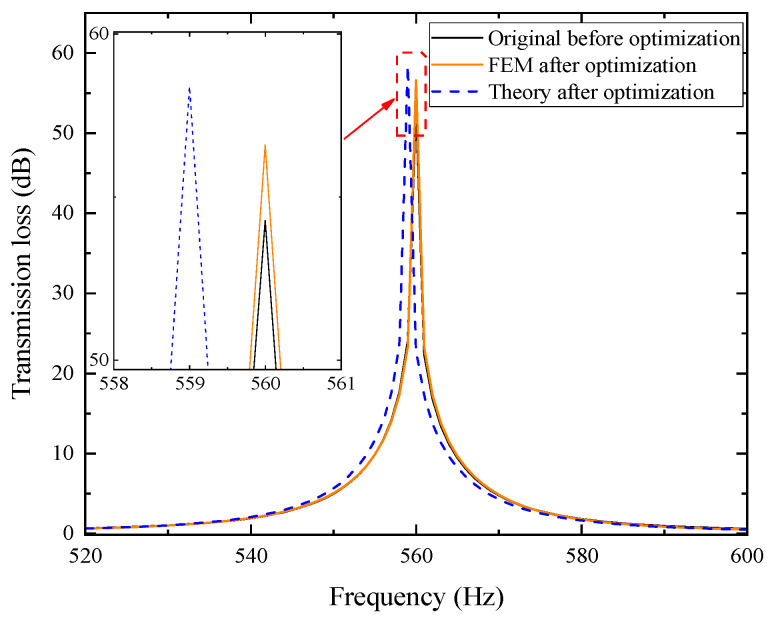
Comparison of noise reduction performance before and after optimization.

After PSO, the final structural parameters of the porous material obtained are *h_dk_* = 31.22 mm and *x_dk_* = 6.21 mm. The optimized finite element model was established using the COMSOL simulation platform, and its sound transmission loss was calculated through theoretical analytical computation. The results before and after optimization were compared, and the curves are shown in Figure 25.

**Figure 26:**
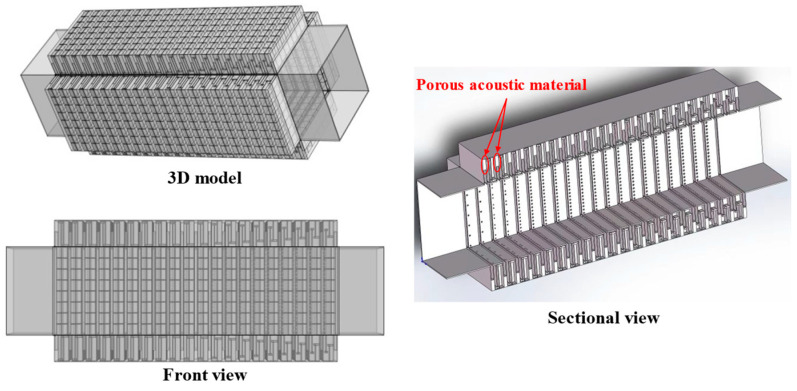
Optimized simulation model of the composite air duct metamaterial muffler.

**Figure 27:**
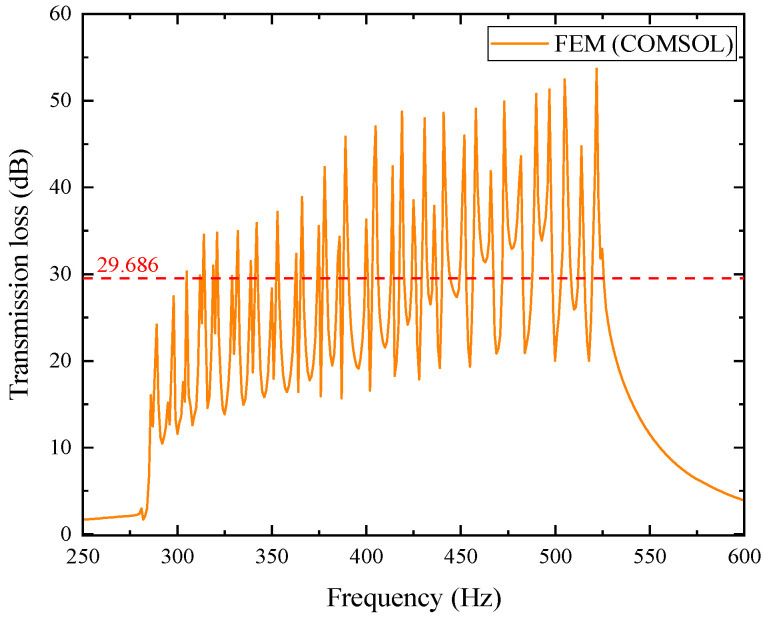
Simulation curve of sound transmission loss for the optimized composite air duct metamaterial muffler.

In the figure, the optimized peak transmission loss has increased to 56.592 dB, which is higher than the 54.275 dB before optimization, and the resonant frequency remains at 560 Hz, indicating a good optimization effect. Subsequently, the 20-layer metamaterial structure without porous materials optimized in Section 5.2 was combined with the optimized porous materials to further improve the overall noise reduction performance. A finite element model of the optimized muffler was established using the COMSOL simulation platform, as shown in Figure 26, and its sound transmission loss was calculated. The results are shown in Figure 27.

**Figure 28:**
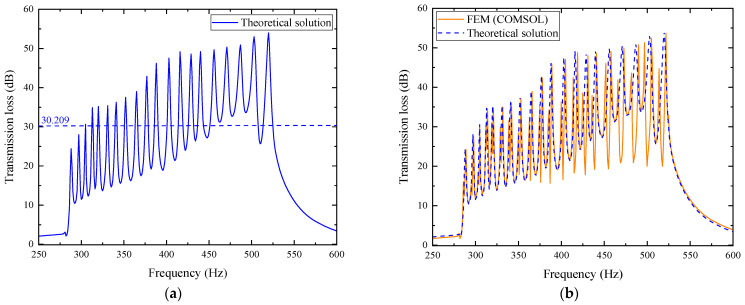
Comparison between simulation and theoretical results of the optimized composite air duct metamaterial muffler: (**a**) theoretical solution; (**b**) comparison of FEM and theoretical solution.

In the figure, the transmission loss of each layer in the composite structure still excites second-order modes, and the effective noise reduction frequency band is slightly extended to higher frequencies, reaching 287~555 Hz, within which the transmission loss is all greater than 10 dB. As the frequency increases, each pair of noise reduction peaks gradually shifts upward. The average transmission loss from the simulation results is 29.686 dB. For further analysis, the theoretical and simulation results are compared, as detailed in Figure 28.

From the comparison chart of optimized simulation and theoretical results, the average transmission loss of the theoretical curve is 30.209 dB, slightly higher than the simulation result of 29.686 dB. In the mid/low-frequency range, the two fit well, and the second-order mode is not obvious; however, in the mid/high-frequency range, the second-order mode is more prominent, leading to a certain deviation between theory and simulation, which affects the average transmission loss.

**Figure 29:**
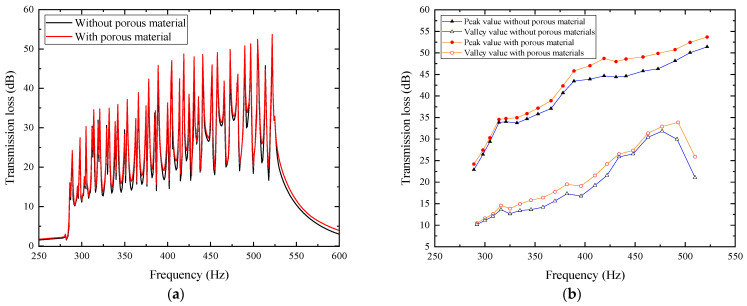
Comparison of sound transmission loss between 20-layer mufflers without and with porous materials: (**a**) frequency characteristics of transmission loss without and with porous materials; (**b**) variation in transmission loss peaks and valleys with frequency without and with porous materials.

To further demonstrate the advantages of the composite porous material structure, the sound transmission loss curves of 20-layer mufflers with and without porous materials are compared. Additionally, the peak values of each layer and the valley values at the joints are plotted for intuitive analysis, with the comparison shown in Figure 29.

In the figure, after adding the optimized acoustic porous material, the overall sound transmission loss has been improved. The peak values of each layer and the valley values at the joints show an insignificant improvement in the low-frequency range, but a significant increase in the mid/high-frequency range, indicating that the porous material has an obvious improving effect on the acoustic performance in the mid/high-frequency range. Compared with the 20-layer structure without porous materials in Section 5.2, the average transmission loss here is 29.686 dB, which has increased by 3.193 dB, about a 12.05% rise. This indicates that the composite air duct metamaterial muffler has achieved the optimal design goal.

## 6. Experimental Validation

### 6.1. Experimental System and Principle

**Figure 30:**
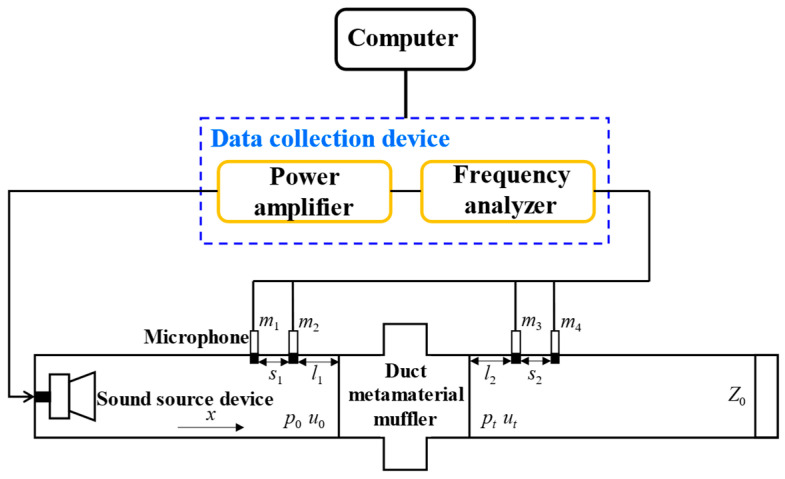
Schematic diagram of the experimental testing equipment for acoustic transmission loss in the impedance tube.

To further verify the accuracy and feasibility of the optimized design in this paper, an experimental test on the transmission loss of the air duct acoustic metamaterial muffler was conducted. Currently, commonly used testing methods include direct measurement methods and indirect measurement methods. This section adopts the four-sensor indirect measurement method to test the muffler sample in an impedance tube, where the front and rear walls of the sample have the same acoustic impedance. The experimental measurement process and the principle of the test system are shown in Figure 30. The testing process includes: first, setting the noise spectrum range and evaluating the signal-to-noise ratio (SNR) to avoid background noise interference; second, adjusting the noise amplitude excited by the sound source at the left end of the impedance tube through a power amplifier; finally, collecting the incident and reflected sound signals at the front and rear ends of the sample using 4 calibrated microphones, and calculating the experimental values of transmission loss based on these signals [37].

The entire experimental testing system includes components such as data acquisition equipment, a power amplifier, an impedance tube, a sound source device, and microphones, as shown in Figure 30. The sound source device is installed at the left end of the impedance tube, and a sound absorption barrier structure *Z*_0_ is arranged at the right end. In the middle, an air duct acoustic metamaterial muffler sample with a thickness of *t* is installed. Two microphone interfaces are arranged on each side of the sample, and the collected signals are transmitted to the computer through the acquisition equipment for analysis and processing.

In the figure, *m*_1_, *m*_2_, *m*_3_, and *m*_4_ are the four microphones; *s*_1_ and *s*_2_ represent the distances between *m*_1_ & *m*_2_ and *m*_3_ & *m*_4_, respectively; *l*_1_ and *l*_2_ represent the distances from *m*_2_ and *m*_3_ to both sides of the sample, respectively. *p*_0_ and *u*_0_ are the sound pressure and velocity near the sound source end in the impedance tube, while *p*_t_ and *u*_t_ are the sound pressure and velocity near the sound absorption barrier structure end. According to the impedance tube design specifications, to ensure that sound waves propagate in the form of plane waves during the test, the length of the sound source end is required to be no less than 3 times the tube diameter. Sound waves are emitted by the loudspeaker and propagate in the tube, forming the incident sound pressure *p*_1_ and reflected sound pressure *p*_2_ at the left end; at the right end, there are the transmitted sound pressure *p*_3_ and the terminal reflected sound pressure *p*_4_. The sound pressures collected by the four microphones are always resolved as plane radiated waves.

The expression for the sound pressure at the left end is:

(30) $$p_{l}\left(\begin{array}{c} x \end{array}\right)=p_{1}e^{jkx}+p_{2}e^{-jkx}$$

The expression for the sound pressure at the right end is:

(31) $$p_{r}\left(\begin{array}{c} x \end{array}\right)=p_{3}e^{jkx}+p_{4}e^{-jkx}$$

The expression for the sound pressure of these four microphones is:

(32) $$\left\{\begin{array}{l} p_{m1}=p_{1}e^{jk\left(s_{1}+l_{1}\right)}+p_{2}e^{-jk\left(s_{1}+l_{1}\right)}\\ p_{m2}=p_{1}e^{jkl_{1}}+p_{2}e^{-jkl_{1}}\\ p_{m3}=p_{3}e^{-jkl_{2}}+p_{4}e^{jkl_{2}}\\ p_{m4}=p_{3}e^{-jk\left(s_{2}+l_{2}\right)}+p_{4}e^{jk\left(s_{2}+l_{2}\right)} \end{array}\right.$$

From the definition of background plane waves, it can be obtained that:

(33) $$\left\{\begin{array}{l} p_{m1}=p_{1}e^{jk\left(s_{1}+l_{1}\right)}+p_{2}e^{-jk\left(s_{1}+l_{1}\right)}\\ p_{m2}=p_{1}e^{jkl_{1}}+p_{2}e^{-jkl_{1}}\\ p_{m3}=p_{3}e^{-jkl_{2}}+p_{4}e^{jkl_{2}}\\ p_{m4}=p_{3}e^{-jk\left(s_{2}+l_{2}\right)}+p_{4}e^{jk\left(s_{2}+l_{2}\right)} \end{array}\right.$$

Thus, the expression for the transmission coefficient *τ* can be obtained as:

(34) $$\tau =\frac{\sin(ks_{1})}{\sin(ks_{2})}\frac{p_{m3}e^{jks_{2}}-p_{m4}}{p_{m1}-p_{m2}e^{-jks_{1}}}e^{jk(l_{1}+l_{2})}$$

Finally, the expression for the sound transmission loss is derived as:

(35) $$STL=20\mathrm{lg}\frac{1}{\left|\tau \right|}$$

**Figure 31:**
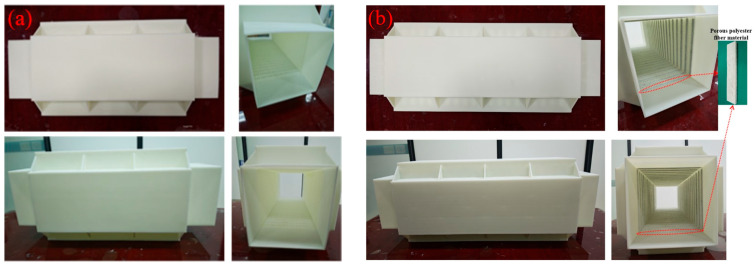
Experimental test samples: (**a**) muffler without porous materials; (**b**) muffler with composite porous materials.

Using 3D printing technology, physical samples of the optimized air duct metamaterial mufflers (both without porous materials and with composite porous materials) were printed using photosensitive resin material, and the 20-layer experimental samples are shown in Figure 31. To enhance structural strength, triangular supporting ribs were added at the four corners of the samples. The dimensions of the entire muffler are 273 mm × 273 mm × 600 mm, and the dimensions of the 20-layer structure on one side are 169 mm × 421 mm × 51 mm. The transmission loss experimental test was conducted on them in accordance with the testing method introduced in the previous statement.

**Figure 32:**
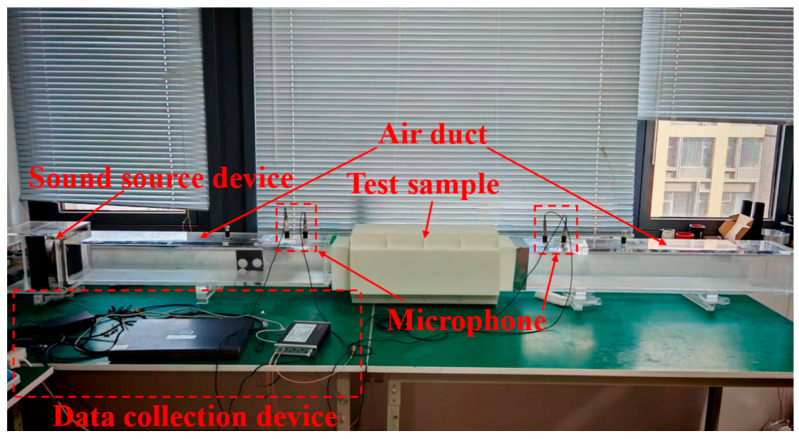
Setup of the experimental test platform.

Among them, the experimental test platform is shown in Figure 32. This test is based on the GB/Z 27764-2011 standard [38], and the BOACH acoustic impedance tube (model: SIT140, manufactured by BOACH ACOUSTICS, Suzhou, China) is used to evaluate the transmission loss characteristics of metamaterial mufflers and composite metamaterial mufflers, with 4 microphones adopted as the evaluation benchmark.

### 6.2. Analysis of Experimental Results

#### 6.2.1. Experimental Results of the Muffler Without Porous Materials

**Figure 33:**
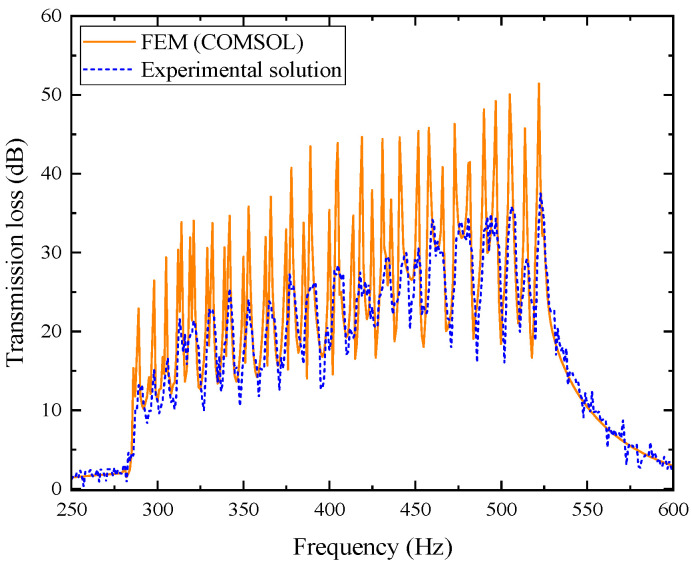
Comparison between experimental and simulation results of the muffler without porous materials.

The experimental sample shown in Figure 31a was placed in the acoustic impedance tube for testing, and the transmission loss curve of the muffler without porous materials was obtained. The experimental results were then compared with the simulation results, as shown in Figure 33.

As shown in Figure 33, the experimental results of the 20-layer metamaterial muffler without porous material show good agreement with the finite element simulations. The transmission loss curves exhibit consistent trends, accurately capturing both the main resonance peak and secondary modes, with the peak positions nearly coinciding, which confirming the accuracy of the model. In the 300~550 Hz range, the experimental curve shows larger fluctuations, mainly due to minor cross-sectional deviations from 3D printing errors and energy leakage caused by imperfect sealing. These factors slightly reduce some peak amplitudes and cause a frequency shift of about 5~10 Hz. Overall, the experimental average transmission loss is 21.739 dB, slightly lower than the simulated 26.493 dB, yet still demonstrating strong low-frequency attenuation capability. This indicates that the model design and FEM predictions are accurate and reliable for characterizing the acoustic performance in the low-frequency range.

#### 6.2.2. Experimental Results of the Composite Muffler with Porous Materials

**Figure 34:**
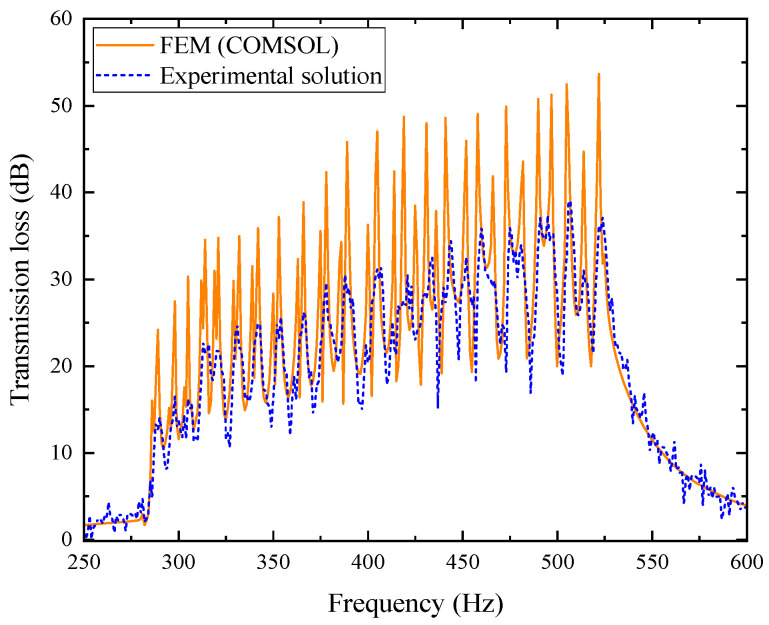
Comparison between experimental and simulation results of the composite muffler with porous materials.

The experimental sample shown in Figure 31b was placed in the acoustic impedance tube for testing, and the transmission loss curve of the composite muffler with porous materials was obtained. The experimental results were then compared with the simulation results, as shown in Figure 34.

As shown in Figure 34, the experimental results of the composite porous metamaterial muffler exhibit a consistent trend with the finite element simulation, accurately capturing the main resonance peak and modal responses. In the primary frequency range (300~550 Hz), both results agree well, indicating that the introduction of the porous layer does not alter the structural resonance characteristics but enhances acoustic energy dissipation. The experimentally measured average transmission loss is 24.599 dB, representing an approximately 13% improvement over the non-porous structure (21.739 dB). The simulated value is 29.686 dB, with the difference mainly attributed to the idealized porous material parameters (e.g., flow resistivity and porosity) and local impedance variations caused by non-uniform polyester fiber density in the test samples. Minor air leakage at the impedance tube joints also leads to slightly lower experimental peaks. Despite these small deviations, the two curves show strong consistency in resonance peak positions and attenuation trends, confirming the reliability of the acoustic model. Particularly in the 320~600 Hz range, the composite structure exhibits a smoother transmission loss distribution, indicating a synergistic energy dissipation mechanism between the porous layer and internal tortuous channels. This significantly enhances sound energy attenuation, demonstrating both improved mid low frequency acoustic performance and strong engineering applicability.

As shown in Figure 33 and Figure 34, the composite porous structure demonstrates superior acoustic performance compared with the non-porous one. The average TL increases from 21.739 dB to 24.599 dB with a smoother curve and broader effective bandwidth. The high flow resistivity of the porous layer enhances viscous dissipation and, together with the internal tortuous resonance, forms a synergistic structural–material damping mechanism, greatly improving low- and mid-frequency attenuation and confirming the superiority and practicality of the composite design.

## 7. Conclusions

Focusing on the challenge of low-frequency broadband noise control, this paper designs a metamaterial muffler structure with perforated and tortuous characteristics incorporating composite porous materials, constructs a new type of composite noise reduction unit, and systematically carries out research work such as theoretical modeling, parameter analysis, optimization design, and experimental verification. The specific research contents are as follows:

(1) An acoustic metamaterial unit with a perforated and tortuous channel structure wrapped with porous materials is proposed. Acoustic models for the perforated plate, tortuous channel, and porous materials are established, respectively, and the theoretical expressions for the total impedance and transmission loss of the composite structure are derived. Verification is conducted using COMSOL simulations, where the theoretical results are highly consistent with the simulation results, thus verifying the accuracy of the model. Subsequently, the effects of types, thicknesses, and widths of porous materials on noise reduction performance are studied, respectively, and it is determined that polyester fiber material has the best performance. The results show that a thickness of 30 mm and a width of 5 mm can significantly improve the transmission loss performance of the muffler, providing a parameter basis for subsequent structural optimization.
(2) Focusing on the arrangement modes of perforated and tortuous metamaterials, the effects of radial and axial periodic distributions on noise reduction performance are analyzed, respectively. It is found that axial periodic arrangement can enhance resonance peaks, while radial arrangement is beneficial to broadening the frequency band. Meanwhile, by axially paralleling multiple unit cell structures with different resonance frequencies, the operating frequency band is effectively expanded, and it is verified that the optimal arrangement order should be resonance frequencies from low to high. A single-layer 32-cell array structure is constructed based on actual air duct conditions, and its mode excitation mechanism in the rectangular air duct is analyzed. It is found that the structure excites first-order and second-order modes at different frequencies, respectively, which further reveals the structure-sound field coupling characteristics.
(3) The PSO algorithm was used to perform parameter optimization on the 20-layer structure without porous materials and the composite porous material structure, respectively. Finally, the average transmission loss was increased from 26.493 dB to 29.686 dB, enhancing the noise reduction capability in the mid- to high-frequency range with an improvement rate of 12.05%. Subsequently, 3D printing technology was used to fabricate the optimized samples, and experimental tests were conducted based on the four-sensor impedance tube method. The results show that there is good consistency among theory, simulation, and experiment. Among them, the average experimental value of the transmission loss for the muffler with composite porous materials reaches 24.599 dB, which is higher than that of the muffler without porous materials, thus proving the superiority of the composite structure.

Compared with traditional straight-channel or expansion-type mufflers, the proposed composite acoustic metamaterial with perforated tortuous channels and externally wrapped porous layers achieves a significant improvement in low-frequency broadband transmission loss. The integration of structural resonance and material damping mechanisms allows an average transmission loss increase from 26.493 dB to 29.686 dB, with an improvement of 12.05%, demonstrating the synergistic effect of the coupled structure. This work also establishes a closed-loop framework that combines theoretical modeling, numerical simulation, parameter optimization, and experimental verification, thus advancing beyond many previous studies that relied solely on simulation. Nevertheless, the model still assumes plane-wave incidence, rigid boundaries, and ideal sealing, which may lead to minor deviations from real conditions. In future work, structural–acoustic coupling and flow–thermal interactions will be incorporated to further enhance predictive accuracy. From an application perspective, the proposed design exhibits good structural compactness, lightweight properties, and manufacturability via 3D printing, indicating strong potential for scaling to practical duct and HVAC noise-control systems, especially in low-frequency broadband ranges where conventional porous materials are less efficient.

## Figures and Tables

**Table 1:** Values of acoustic parameters for a polyester fiber porous material.

Porosity	Flow Resistivity	Tortuosity Factor	Viscosity Characteristic Length	Thermal Characteristic Length
92%	70,590 Pa·s/m^2^	1.58	2.678 × 10^−5^ m	6.42 × 10^−4^ m

**Table 2:** Acoustic parameter values of a certain rock wool porous material.

Porosity	Flow Resistivity	Tortuosity Factor	Viscosity Characteristic Length	Thermal Characteristic Length
94%	135,000 Pa·s/m^2^	2.1	4.9 × 10^−5^ m	1.66 × 10^−4^ m

**Table 3:** Acoustic parameter values of a certain glass wool porous material.

Porosity	Flow Resistivity	Tortuosity Factor	Viscosity Characteristic Length	Thermal Characteristic Length
99%	9000 Pa·s/m^2^	1.0	19.2 × 10^−5^ m	38.46 × 10^−5^ m

**Table 4:** Noise reduction performance of acoustic metamaterials with different resonant frequencies.

Serial Number of Unit Cell Structure	Number of Perforations	Perforation Diameter (mm)	Resonance Peak Frequency (Hz)	Transmission Loss Peak (dB)
Q	1	2	348	34.976
W	2	2	362	47.213
E	3	3	376	38.298

**Table 5:** Characteristic values of sound transmission loss curves for two arrangement orders.

Arrangement Form	TL Peak 1; Corresponding Frequency	TL Peak 2; Corresponding Frequency	TL Peak 3; Corresponding Frequency
Q, W, E	35.051 dB; 348 Hz	49.756 dB; 362 Hz	49.622 dB; 376 Hz
E, W, Q	30.315 dB; 348 Hz	43.190 dB; 362 Hz	48.089 dB; 376 Hz

**Table 6:** Optimization frequency ranges for each group.

Group	Frequency Interval Range (Hz)
1	$\left[290\pm 2,\;320\pm 2\right]$
2	$\left[330\pm 2,\;380\pm 2\right]$
3	$\left[390\pm 2,\;440\pm 2\right]$
4	$\left[450\pm 2,\;520\pm 2\right]$

**Table 7:** Optimized structural parameters (mm).

Layers	$H_{1}$	$H_{2}$	$v_{L}$	$w_{R}$	$y_{1}$	d
1	45.96	45.91	4.50	9.01	9.49	2.98
2	45.77	45.63	4.41	8.93	9.42	3.04
3	45.83	45.81	4.38	8.87	9.39	2.21
4	45.81	45.76	4.36	8.86	9.37	2.55
5	45.84	45.87	4.40	8.88	9.36	2.68
6	45.73	45.71	4.36	8.81	9.33	3.12
7	45.68	45.58	4.27	8.77	9.28	3.13
8	45.33	45.21	4.07	8.66	9.15	3.18
9	44.72	44.94	3.89	8.63	9.08	3.19
10	44.23	44.21	3.73	8.49	8.96	3.24
11	43.86	43.74	3.68	8.31	8.73	3.25
12	42.43	41.77	3.55	8.29	8.62	3.30
13	40.91	40.61	3.43	8.13	8.50	3.33
14	39.69	38.62	3.31	8.02	8.39	3.41
15	38.22	37.13	3.19	7.89	8.27	3.43
16	35.87	36.59	2.95	7.75	8.15	3.47
17	34.93	33.86	2.78	7.63	7.98	3.58
18	33.65	31.57	2.66	7.49	7.74	3.64
19	32.46	30.20	2.42	7.25	7.44	3.71
20	31.11	28.97	2.28	6.88	7.32	3.92

## Data Availability

The original contributions presented in this study are included in the article. Further inquiries can be directed to the corresponding authors.
